# Supplementary material for: Identification of d-arabinan-degrading enzymes in mycobacteria
Source: Nat Commun. 2023 Apr 19;14:2233. doi: 10.1038/s41467-023-37839-5 (PMC10115798; doi:10.1038/s41467-023-37839-5)
Supplement: Supplementary file 1 — Supplementary Information [file 41467_2023_37839_MOESM1_ESM.pdf]

Supplementary Information:
Identification of D-arabinan-degrading enzymes in mycobacteria

Supplementary Figures

Figure S1: Identification of PULs associated with D-galactan degradation
Figure S2: *D. gadei* whole cell proteomics
Figure S3: TLC of GH172 enzyme catalysed reactions.
Figure S4: Michaelis-Menten Kinetics of GH172 enzymes against pNP-α-D-Ara*f* and AG.
Figure S5: SEC-MALs of GH172 enzymes
Figure S6: Sequence alignment of GH172 enzymes
Figure S7: SEC-MALS analysis of Dg_{GH172c} mutants
Figure S8: Phylogeny of DUF4185 enzymes found in selected acid-fast bacteria
Figure S9: Organisation of phage lysis cassettes in cluster C, DQ and a *Rhodococcus* phage singleton
Figure S10: TLC of DUF4185 enzyme catalysed reactions.
Figure S11: Sequence alignment of DUF4185 proteins
Figure S12: Start site analysis for Rv3707c
Figure S13: Release of fluorescently labelled D-arabinan by selected acid-fast enzymes
Figure S14: Uncropped TLCs for figures in this paper.

Supplementary Tables

Table S1: Recipe for 2x defined minimal media

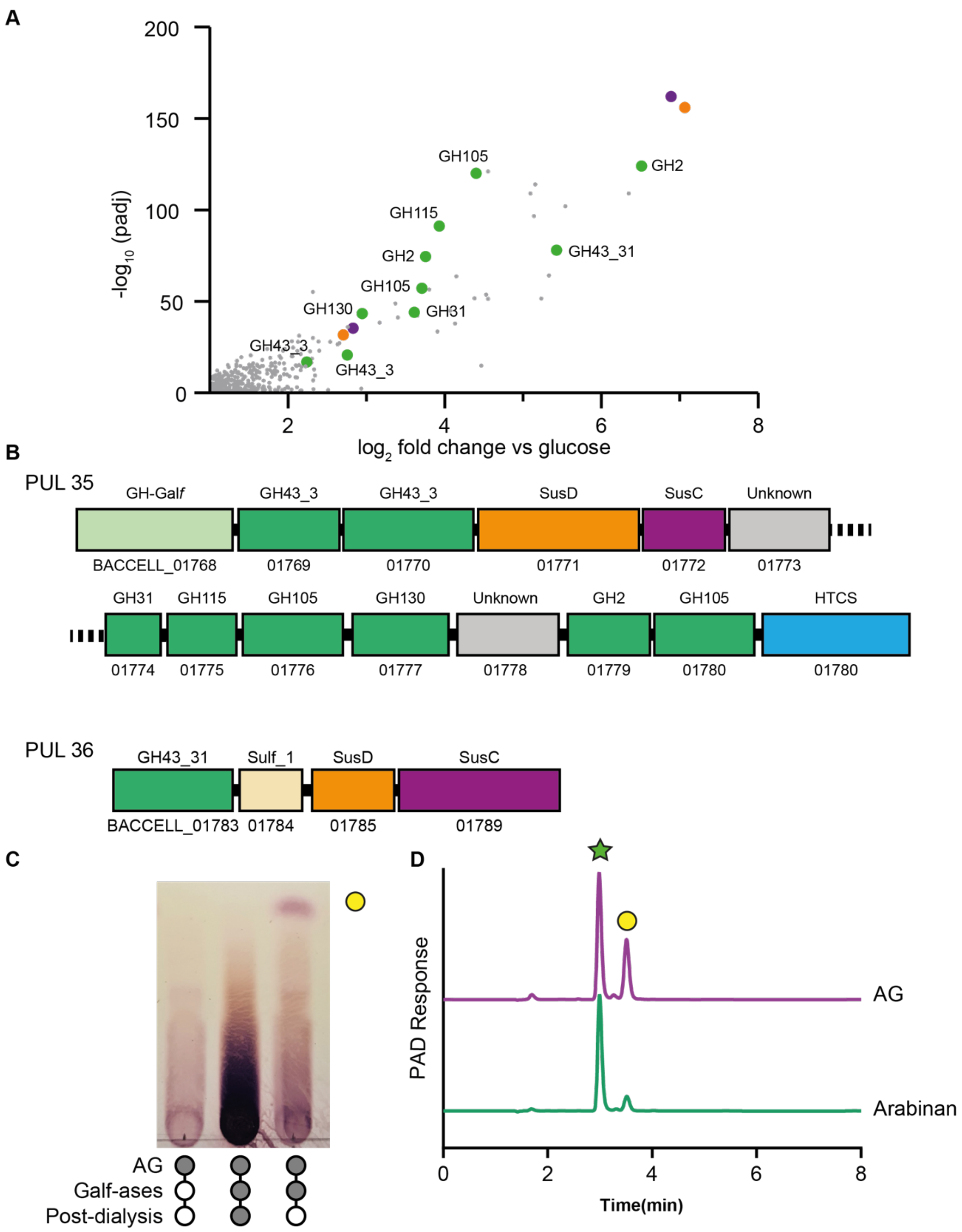

Figure S1. Identification of PULs associated with D-galactan degradation. A) Transcriptomic analysis of *B. cellulosilyticus* cells grown on arabinogalactan (AG) as compared to those grown on glucose as a sole carbon source. Transcriptomic analysis of B. cellulosilyticus cells grown on AG (n=3) as compared to those grown on glucose (n=3) as a sole carbon source. Differentially expressed genes were determined according to a negative binomial model with DESeq2 and adjusted for multiple testing using the Benjamini-Hochberg method. **B)** PULs identified as being upregulated during growth on AG based on the proteomic analysis. **C)** AG from *M. smegmatis* was treated with 1 μM *B. finegoldii* enzymes BACFIN_08810 and BACFIN_04787 overnight, then dialysed to remove free galactose. **D)** Acid hydrolysed aliquots of treated arabinan and untreated arabinogalactan were analysed by ion-exchange chromatography with pulsed amperometric detection (IC-PAD). Comparison to arabinose and galactose standards showed an approximate 70% reduction in galactose in the treated D-arabinan. Green star – D-arabinose, yellow circle – D-galactose. Source data are provided in the Source Data file.

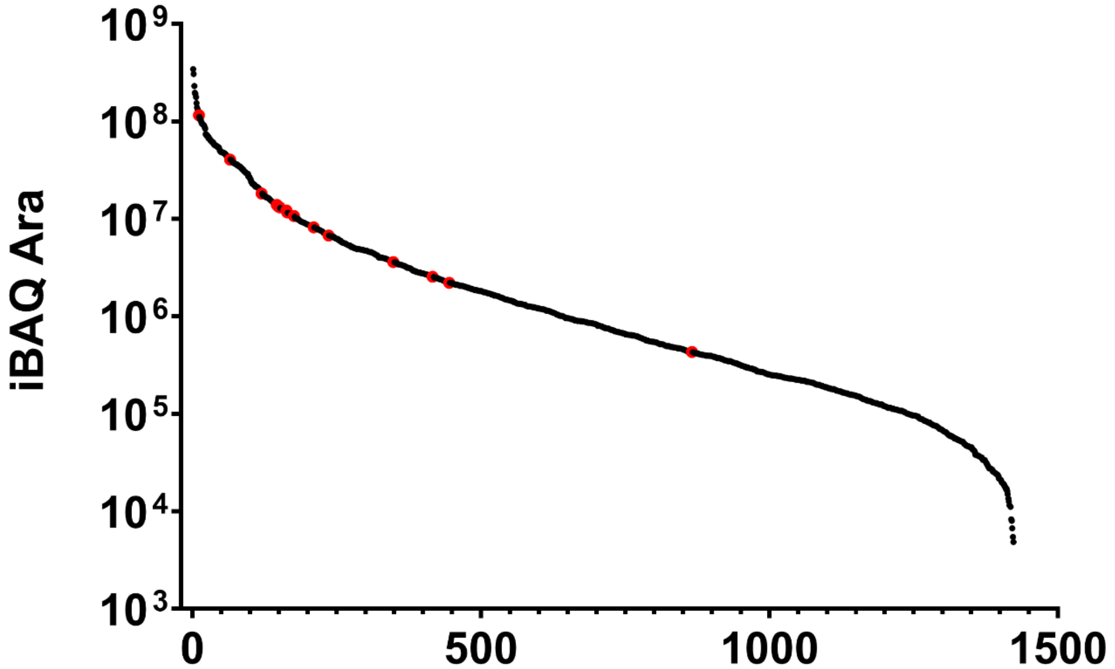

Figure S2: *D. gadei* whole cell proteomics Dynamic range of the *D. gadei* proteome, based on ranked iBAQ intensity, when grown on D-arabinan. Proteins from PUL42 are identified in red. The mass spectrometry data have been deposited to the ProteomeXchange Consortium via the PRIDE partner repository with the dataset identifier PXD039984 (http://www.ebi.ac.uk/pride/archive/projects/PXD039984).

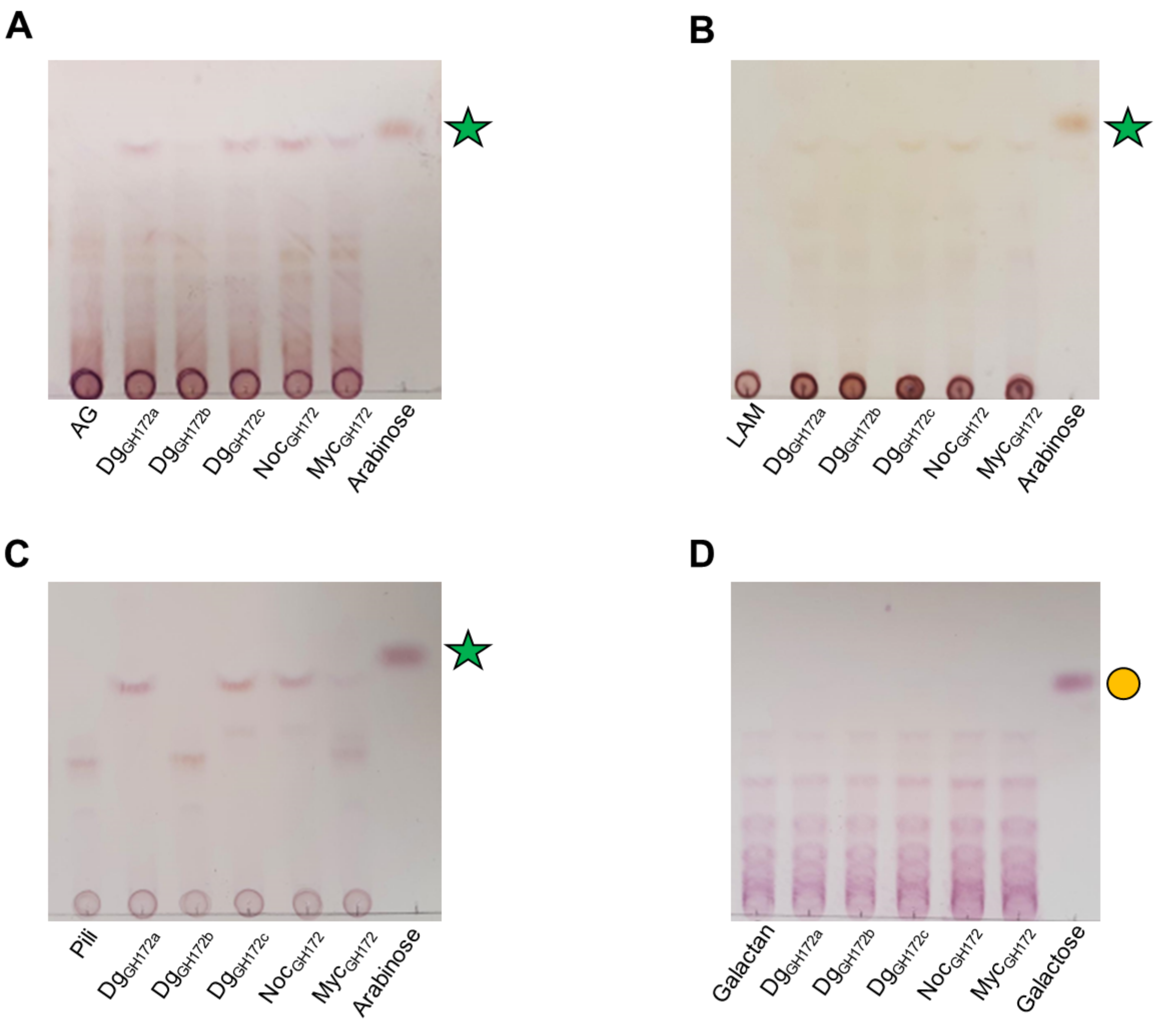

Figure S3: TLC of GH172 enzyme catalysed reactions. GH172 enzymes (Dg$_{GH172a}$, Dg$_{GH172b}$, Dg$_{GH172c}$, Noc$_{GH172}$, and Myc$_{GH172}$) were incubated with arabinogalactan (**A**), LAM (**B**), pilin oligosaccharides (**C**), and galactan (**D**) overnight at 37 °C, analysed by TLC and stained with orcinol. Yellow circles = D-galactose, green stars – D-arabinose.

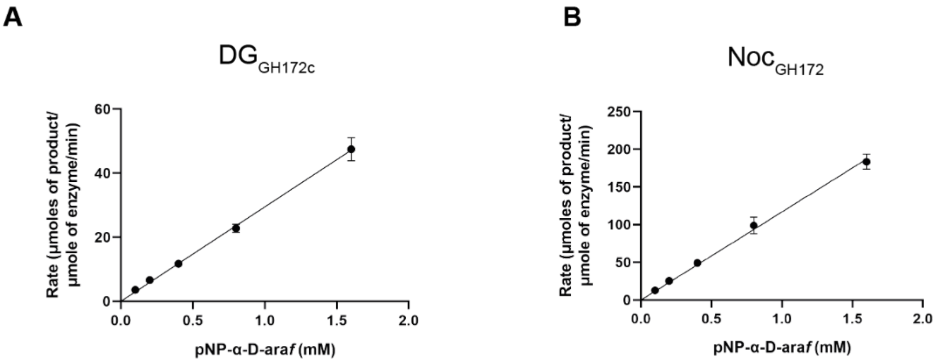

Figure S4: Michaelis-Menten Kinetics of GH172 enzymes against pNP-α-D-Ara*f*. Rates were measured with 100 nM enzyme at 37 °C in 20 mM HEPES pH 7.5, against varying concentrations of p-nitrophenyl-α-D-Ara*f* at 400 nm. Each concentration was repeated in triplicate, in some cases the error bars fall within the plotted data-points and are not visible in the plots. n = 3 biological replicates, error bars are +/- standard deviation. Source data are provided in the Source Data file.

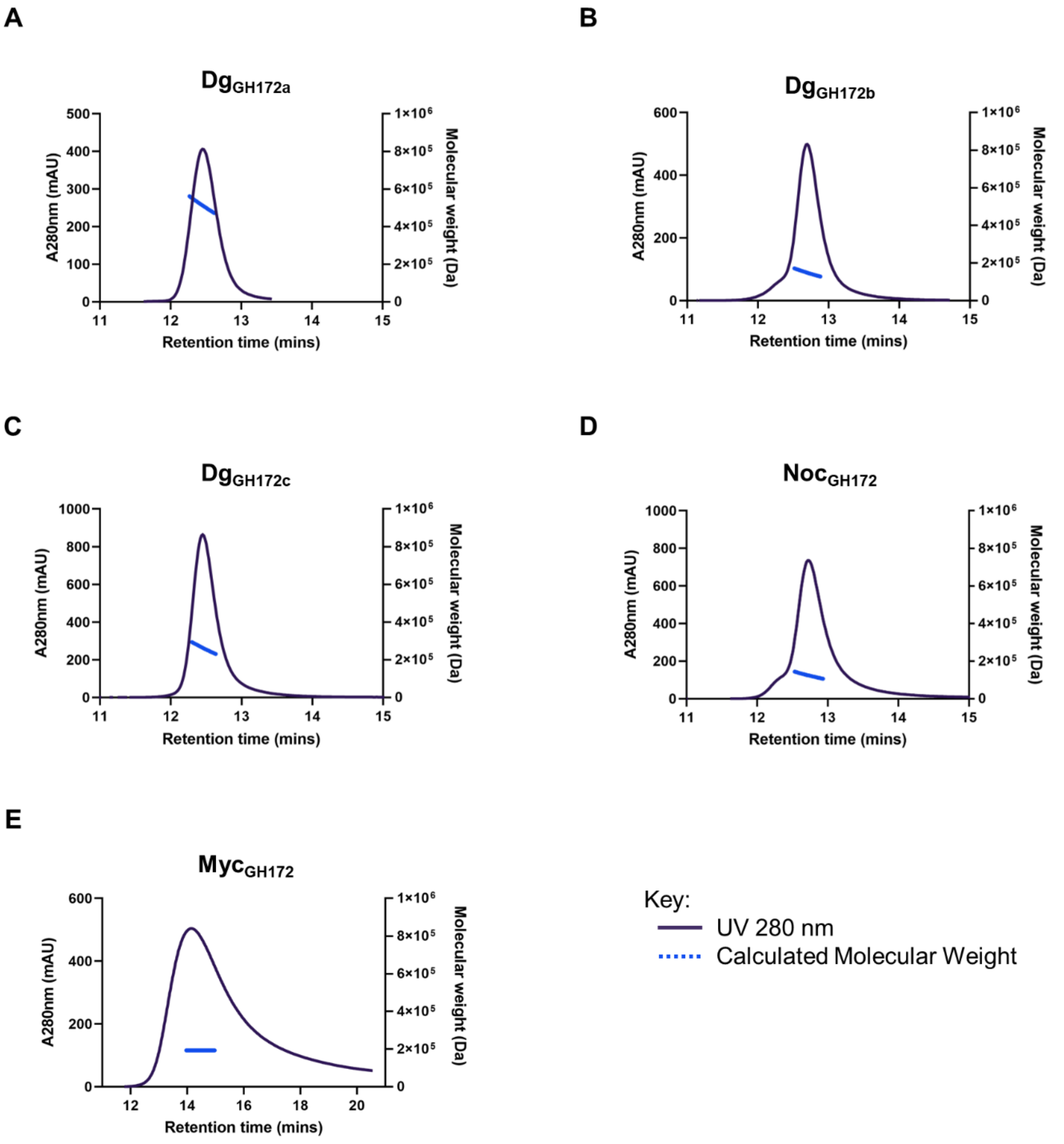

Figure S5: SEC-MALs of GH172 enzymes. SEC-MALS analysis of Dg$_{GH172a}$ (**A**), Dg$_{GH172b}$ (**B**), Dg$_{GH172c}$ (**C**), Noc$_{GH172}$ (**D**), and Myc$_{GH172}$ (**E**) allowing assignment of average masses to each GH172 enzyme (**Table 3**). Masses are consistent with: Dg$_{GH172a}$ being a dodecamer; Dg$_{GH172b}$ as a dimer species; Dg$_{GH172c}$ as a hexamer; and Noc$_{GH172}$ and Myc$_{GH172}$ as trimeric species. Source data are provided in the Source Data file.

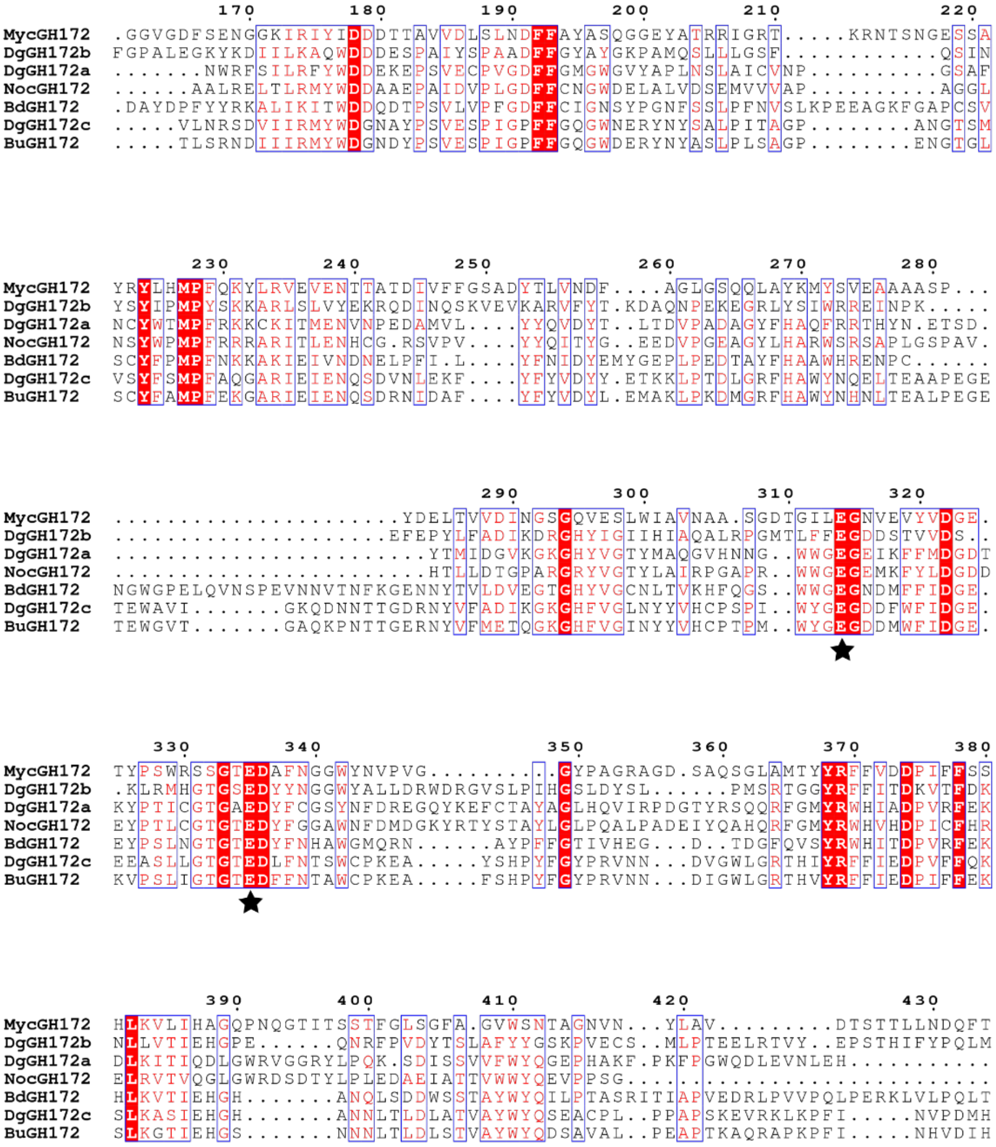

Figure S6: Sequence alignment of GH172 enzymes. Protein sequence alignment of GH172 enzymes (Dg_{GH172a}, Dg_{GH172b}, Dg_{GH172c}, Bu_{GH172}, Bd_{GH172}, Noc_{GH172}, and Myc_{GH172}). Catalytic residues have been highlighted by a black star. Red boxes with a white character show strict identity, and red character with blue frames show similar residues. Protein sequences were sourced from Uniprot and alignment was performed using Clustal Omega, and then formatted with ESPript 3.

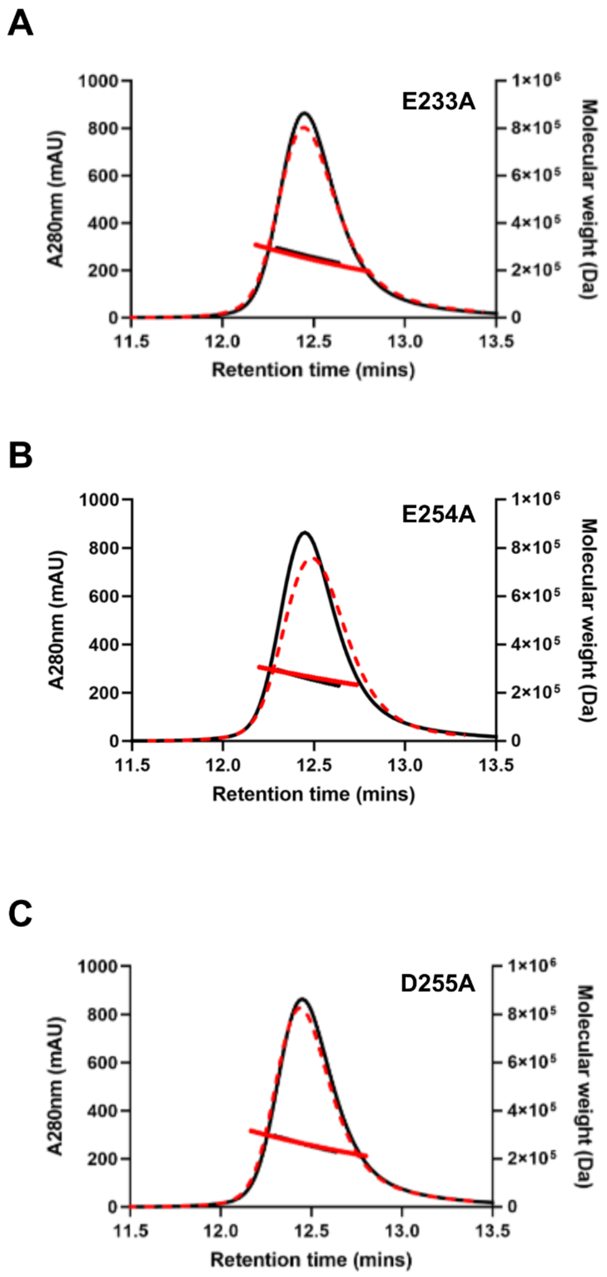

Figure S7: SEC-LS analysis of Dg_{GH172c} mutants. SEC-MALS analysis of Dg_{GH172c}-E233A (**A**, dashed red line), Dg_{GH172c} -E254A (**B**, dashed red line) and Dg_{GH172c} -D255A (**C**, dashed red line) overlaid with Dg_{GH172c}-WT (black line, **A**/**B**/**C**). All mutants have a weight consistent with that of a hexamer and similar to WT. Source data are provided in the Source Data file.

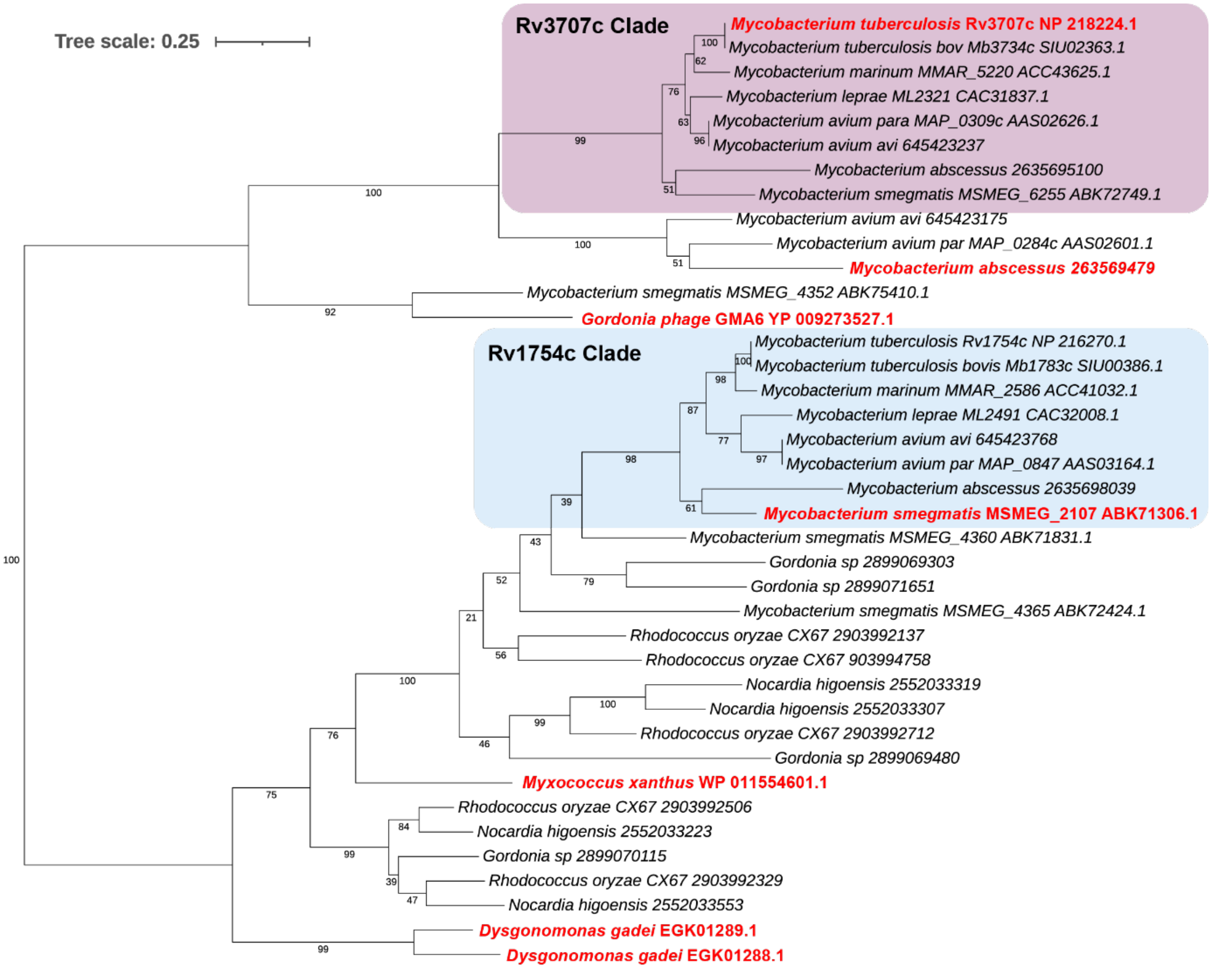

Figure S8: Phylogeny of DUF4185 enzymes found in selected acid-fast bacteria. A restricted alignment of 39 complete sequences from a selection of acid-fast bacteria and enzymes characterised in this study were aligned with Clustal Omega using default settings in SEAVIEW v.4.6.4 with some manual adjustments[58,59]. The alignment shows two clear clades of DUF4185 enzymes in acid fast bacteria with some further diversification, mainly in non-mycobacterial species.

.

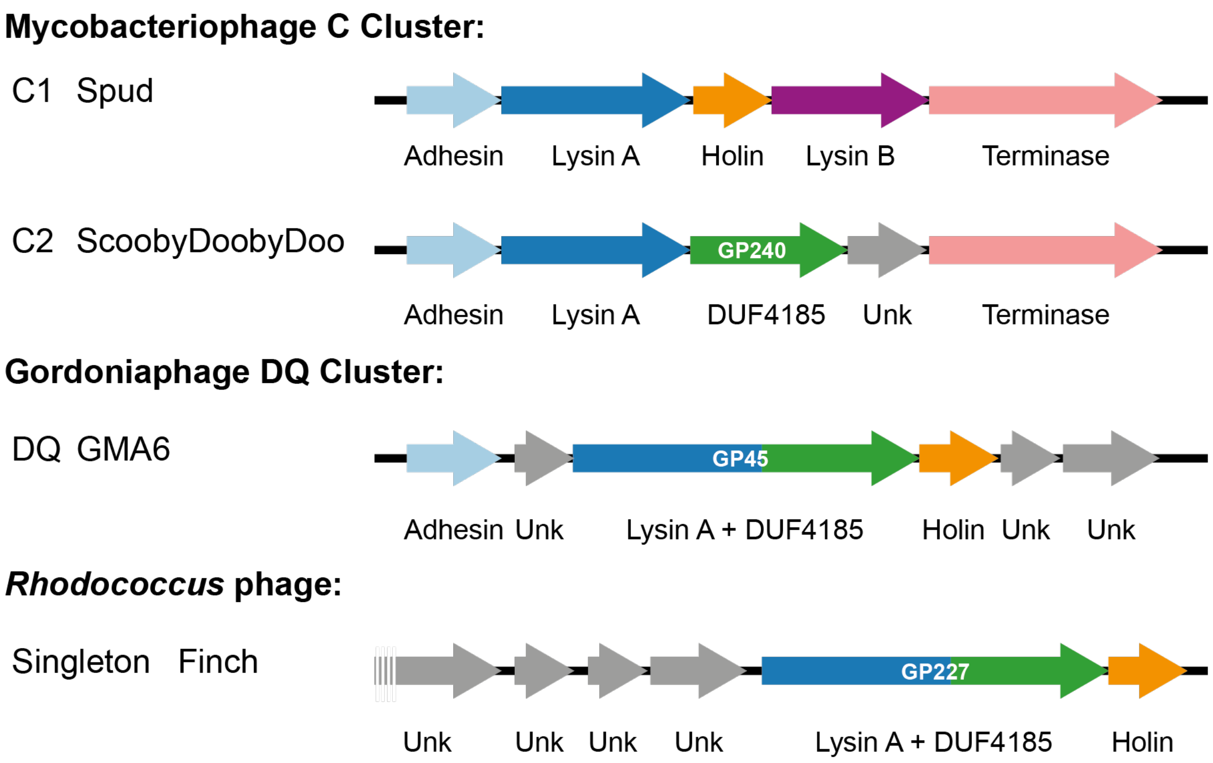

Figure S9: Organisation of phage lysis cassettes in cluster C, DQ, and a *Rhodococcus* phage singleton. Genomic regions encoding the lysis cassettes for clusters C1, C2, DQ in addition to the singleton Finch. Lysin B is absent in C, DQ and Finch and appears to be replaced by either a separate DUF4185 enzyme or as a LysinA-DUF4185 fusion. Unk – unknown.

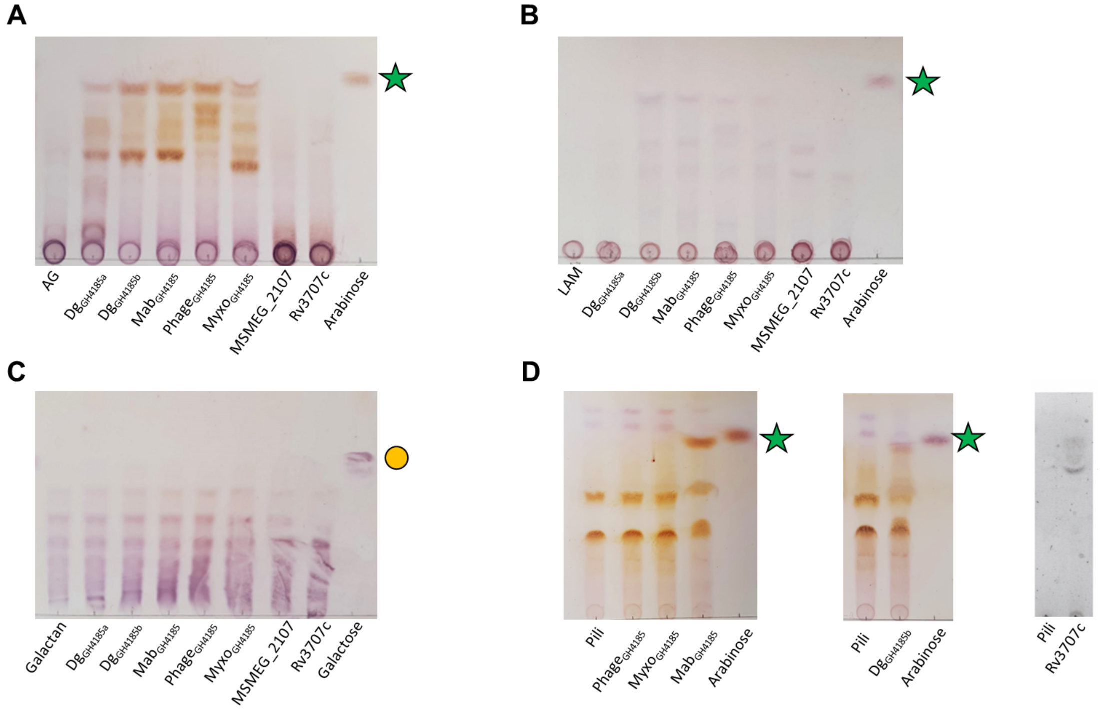

Figure S10: Thin-layer chromatography (TLC) analysis of DUF4185 enzyme catalysed reactions. DUF4185 enzymes ($Dg_{GH4185a}$, $Dg_{GH4185b}$, Mab_{GH4185}, $Phage_{GH4185}$, $Myxo_{GH4185}$, MSMEG_2107, and Rv3707c) were incubated with arabinogalactan (**A**), LAM (**B**), galactan (**C**), and pilin oligosaccharides (**D**) overnight at 37°C. 10 µL of each reaction was spotted at the origin of the TLC and the TLC was developed in a system consisting of 2:1:1 (v/v) of 1-butanol:acetic acid:water and visualised by staining with orcinol and charring. Yellow circles = D-galactose, green stars – D-arabinose.

```
             170       180       190        200       210       220
MSMEG_2107   TDLGIMWDNGN.PGARQVLMAFGDTYGY..C......GVRGQQWRYNVLFRSQDGALSNTIAVPNGV
Rv1754c      TDLGIPWDNGD.PANRQVLMIFGDTFGY..C......AVDGHQWRYNTLFRSQDRDLGNGVHVTSGD
Myxo4185     TDLGIVWDKGG....GEVFVLFGDTFGAGWCGN....GGCGGGWRSNVLARSSDTNLANGLSFSSM.
DgGH4185a    TDLGIVWEMNQ....GKYGIFLGDTFGKGFIPDQNDPADFGDNWRCNVLAFSNDCNLEDGLSIDSM.
DgGH4185b    TDLGIIWEMNP....GKYGIFFGDTFGRDFSPNSAAPGPNGGSWRSNVLAFSDDNDLEDGLTFNSM.
Rv3707c      TDLCEFVEFPS.....QLLQVCGDSFAGQGVGF...GG.....WYAPVALHVDT.ES.....IDDP.
MabGH4185    ADIGEVIEIPDGKGGKKLVAVFGDSFSTDHVPR...PGEAADHYKSVA.VEIKG.FDQNGKPIWGD.
PhageGH4185  TDLGAMIEAPNG....KLVAVFGDTWKDRVGG.........IDWRAPV.ILIGS.KGANGVIQWER.
              ★                             ★

               230       240       250             260       270      280
MSMEG_2107   PRNKFSGSPVWAPGISKQIINSTKW..AATEKGIIPTA..GVAVGQTQYLNFMSIRNWDSDGRWSTN
Rv1754c      ASNRYSGSPVRQPGFSKQLINSIKW..ARDETGIIPTA..GIAVGKTQYVNFMSIRNWGRDGEWTTN
Myxo4185     ........IQDGPRHAKEILASRKV..NFDEITVIPTA..GVTIGSRHYIHYMSIHHWGEAGMWFTN
DgGH4185a    ........ASDENDNARELIPGGKDKSGNGDWTSIPTA..AIRANNKDYVHYFNMRNWT...GWITN
DgGH4185b    ........AVDVSGNAREIIYGGKDASGNGDWTSIPTA..AIRANNIDYVHYFNMKNWT...GWITN
Rv3707c      AGVRYTGVTGVGTPLLAD..PTPPG......DSQLPAGVVQIN.RRNYLMVTTT.....KDLQPQNS
MabGH4185    VLTGYDGENGRPPLFPTT..GLPPA.AQNT..NTLPAGSIVMRDGTTYMMAAGT.....NDLHP...
PhageGH4185  AG.GSDPNYARQLWAYTH..DSPPW.TKGGISTVLPS..DLLRIGNTLYLHVMV.....NKGFPTVA

             290       300       310       320       330           340       350
MSMEG_2107   YSAIAVSPDNGENWGVYPGTVRTPADGVVSGARYVRGNENFQQGAFLKP....GPGDPYLYTFGTPS
Rv1754c      YSGIAVSKDNGQTWGVFPGTIRASGPDSGGKARFVPGNENFQMGAYLKS....N..DGYLYSFGTPP
Myxo4185     HAGIAYSDDQGQNWVKHPTARWQNTPA.........WTNNFQMAAFVRN.......GGFVYMYATPN
DgGH4185a    YSGLYKSEDDGKTWGKCTTVHFTSD............SNFGQAGYYKK.......DGYVYMVGTET
DgGH4185b    YSGMYKSIDNGQTWAKCENVRFSSN............SNFGQGGYFKK.......DGYVYMIGTQT
Rv3707c      ..RLVRAEAARGGWQTVSGSRRNAAY.........QDGRQTQISGYYDPVPTPDSPTGWVYIVADSF
MabGH4185    .........TGGSWLVKAGDPSQGGWPPEPGTWRAGDKAPSQISGY.......QAADGTVYIAADSF
PhageGH4185  WTEIWKSEDSGISWTHLGENSKLS......GTA.GNGLLQCLSWDY.......NADDDYVYAVSTGF

                   360       370       380       390       400       410
MSMEG_2107   GRGGAAYIARVAPGA.VPDLNRYEYWNSNTN.SWVPRDPGAATPVIPGPVGELSAQYNTYLKQYVVL
Rv1754c      GRGGSAYLARVPQRF.VPDLTKYQYWNGDSN.SWVPNKPDAATPVIPGPVGEMSVQYNTYLKQYLAL
Myxo4185     GRFGNVHLARVPETA.LLDINGYRYWDGN...GWSVSQ.AAARPVAIGIAGELSVSYHAPLGRFLMT
DgGH4185a    GRHSNPRLARFNEKD.IENQSLYEYWNKDHN.EWIRGDESQATNLFEDTVGELSIAYHNKYKCWIIT
DgGH4185b    GRDSNARLARFKEQH.IEDQANYEFWNSLTK.QWLKGDEEQATNIFNDKVGELSFIYNQKHNKWIIS
Rv3707c      TRGEPAVLYRATPES.FTDRSRWQGWAGGPDGGWNKPPTP....LWPDQLGEMSIR..QIDGQTVLS
MabGH4185    DRNQQVTMYRVPPGGNVLDRDSWQ...PMTNTGWGAPGDPAK.PLTTTPYGEVSLR..EVDGRPVLS
PhageGH4185  QRDKGIVLRRFRPTQ.INNQSLWQVWNYNGTWGWRTNVNPSVITPSGEKWGELTLR..KLANGWVLG

               420       430                 440       450       460
MSMEG_2107   YCN.GANDVVMRTAQAPQG..........PWGPE......QLLVPSTQYPGGIYAPFLHPWS.TGKE
Rv1754c      YTN.GMNDVVARTAPAPQG..........PWSAE......QMLVSSWQMPGGIYAPMMHPWS.TGKD
Myxo4185     YLNEHRQAVVMRDAATPTG..........PWSGE......KVLASGAVYP.GLYNAFIHPWGNTGNS
DgGH4185a    YFCAARYNISIRYAEELTG..........QWSKP......QILATGEEYP.QLYGSYIHPVSLDGDD
DgGH4185b    YFNADRYNITMRTATEITG..........PWSEP......YELASGVEYA.QLYGSYFHPLSVDGDN
Rv3707c      YFNASTGNMEVRVAHHPT.SLGAAPVTTVVRHDEWPEPAESLPPPYDNRLAQPYGGYISPGSTIDE.
MabGH4185    GLNMGPDPGNGTARVEVRVGSAGDPSSVFAWNSPATVLMQQADPTAPNFVLQNYGGYILPGSTLDN.
PhageGH4185  GLSMHSLGLSYRVLSSPTANLYTTPAKRLIWGCEW.....PEEDHTVGKVAQTYGGYIVPGSKLGVD

                470       480
MSMEG_2107   ..LYFNLSLWS.......AYNVMLMRTVLP...............
Rv1754c      ..VYFNLSLWS.......AYNVMLMHTVLP...............
Myxo4185     ..LYFVMSQWT.......PYNTFLMRATLAHD.............
DgGH4185a    ..LYFLMSMWR.......PYNVFLMRAELELEHHHHHH.......
DgGH4185b    ..LYFLMSMWL.......PYNVFLMKAELADMGSFDLEHHHHHH.
Rv3707c      ..LRIFVSQWDTRARQNGPYRVIQFAVNPFKPWSDPGSGHHHHHH
MabGH4185    ..MRVFGSQWVVAD..GTPYNTQLIEVNPHQ..............
PhageGH4185  GGVDIVVSQWNTTV..GWPYKVMQFRGTL................
```

Figure S11: Sequence alignment of DUF4185 proteins

Protein sequence alignment of DUF4185 enzymes with putative active site residues highlighted with black stars. Red boxes with a white character show strict identity, and red character with blue frames. Sequence aligned using Clustal Omega and visualised using ESPript.

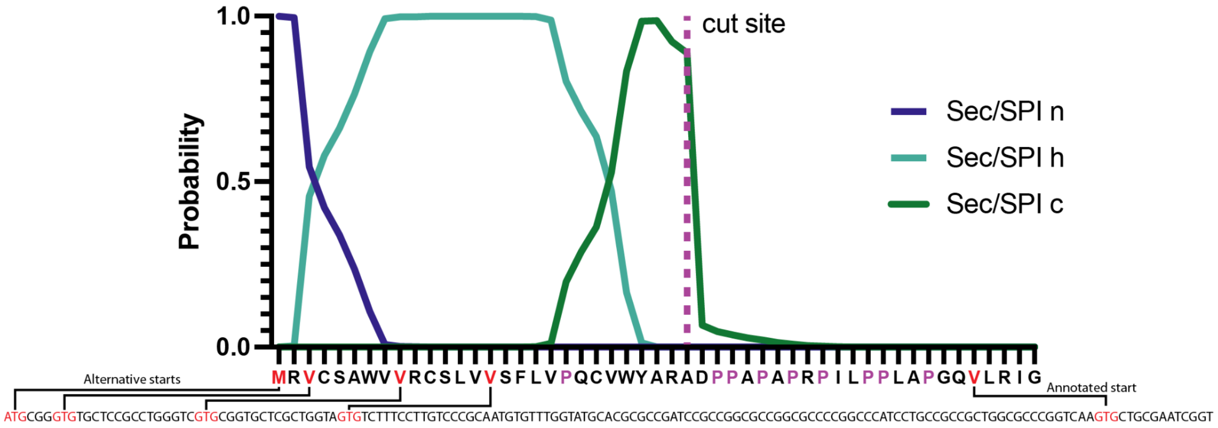

Figure S12: Start site analysis for Rv3707c. SignalP 6.0 output for Rv3707c with four potential upstream start sites included. Proline residues are indicated in purple.

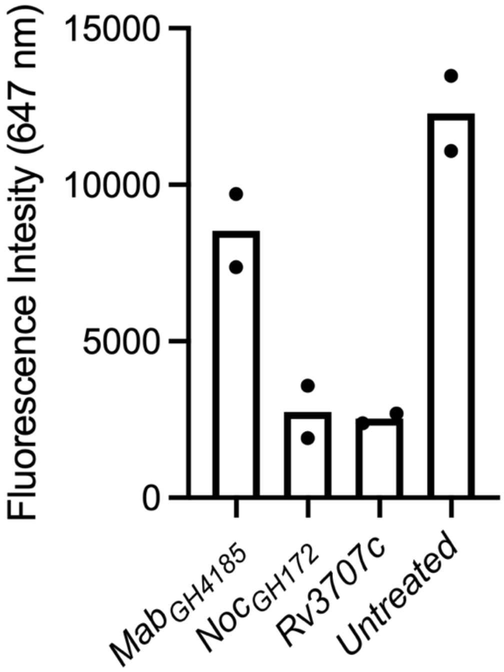

Figure S13: Release of fluorescently labelled D-arabinan by selected acid-fast enzymes. Fluorescent mycolyl-arabinogalactan-peptidoglycan complex (mAGP) obtained from 5-AcFPA treated *C. glutamicum* was incubated with 1 µM of each enzyme with rotation overnight. After 3 washes, the remaining fluorescent signal was measured. Enzyme activity is represented as a reduction in fluorescence relative to the untreated control (n = 2 technical replicates). Source data are provided in the Source Data file.

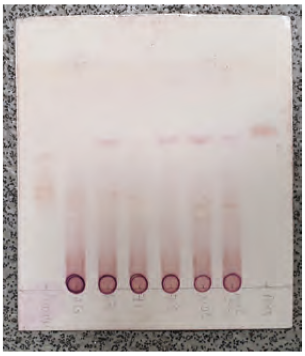

Figure S3A

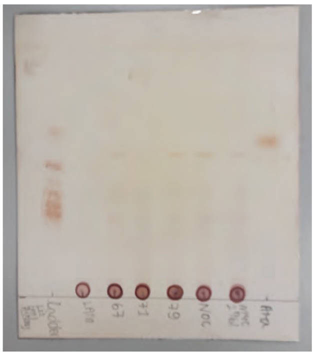

Figure S3B

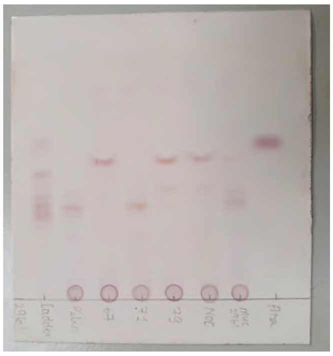

Figure S3C

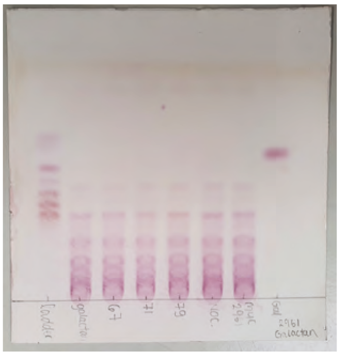

Figure S3D

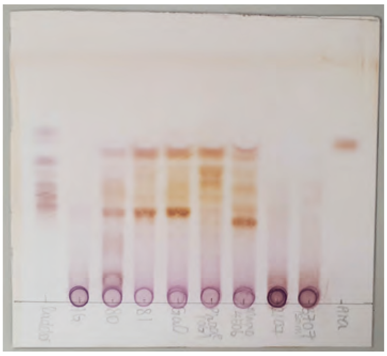

Figure S10A

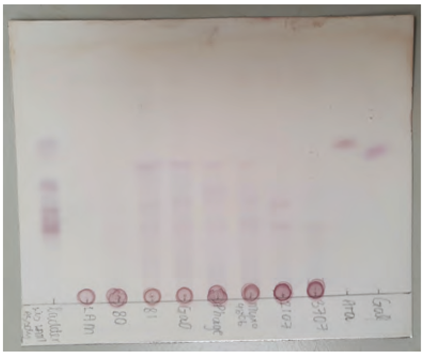

Figure S10B

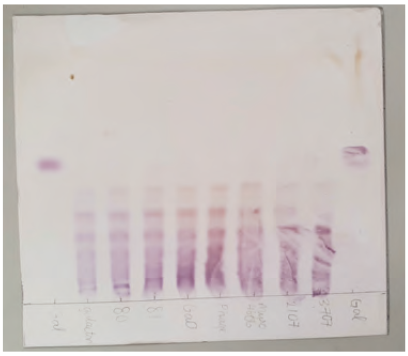

Figure S10C

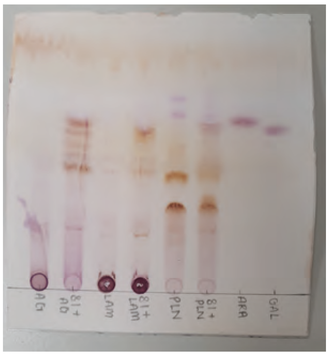

Figure S10D

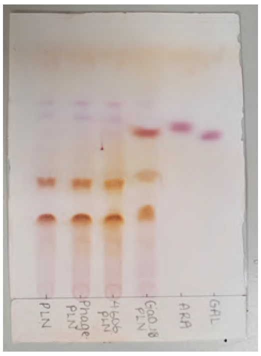

Figure S10D

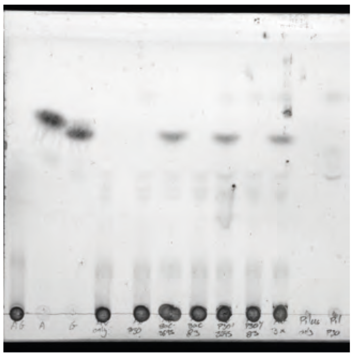

Figure S10D

Figure S14: Uncropped TLCs for figures in this paper.

Supplementary Tables

Table S1: Recipe for 50ml of 2x defined minimal media

Component	Mass (mg) or volume (ml)
**10x Bacteroides salts (4L)**<br>(544g KH_2PO_4, 35g NaCl ,45g $(NH4)_2SO_4$ – pH 7.2)	10 ml
**Balch's vitamins (1L)**<br>(p-Aminobenzoic acid, 5mg<br>Folic acid, 2mg<br>Biotin, 2mg<br>Nicotinic acid, 5mg<br>Calcium pantothenate, 5mg<br>Riboflavin, 5mg<br>Thiamine HCl, 5mg<br>Pyridoxine HCl, 10mg<br>Cyanocobalamin, 0.1mg<br>Thioctic acid, 5mg<br>Distilled Water, 1L)	1 ml
**Trace mineral solution (1L)**<br>EDTA, 0.5g<br>$MgSO_4$*7H_2O, 3g<br>$MnSO_4$*H_2O, 0.5g<br>NaCl, 1g<br>$FeSO_4$*7H_2O, 0.1g<br>$CaCl_2$, 0.1g<br>$ZnSO_4$*7H_2O, 0.1g<br>$CuSO_4$*5H_2O, 0.01g<br>H_3BO_3, 0.01g<br>Na_2MoO_4*2H_2O, 0.01g<br>$NiCl_2$*6H_2O, 0.02g	1 ml
**Purine/Pyrimidine solution (1L)**<br>200 mg each: Adenine (Sigma, A2786)<br>Guanine (Sigma, G11950)<br>Thymine (Sigma, T0895)<br>Cytosine (Sigma, C3506)<br>Uracil (Sigma, U1128)	1 ml
**Amino acid solution (250 ml)**<br>62.5 mg of all 20 standard amino acids	1 ml
Vitamin K3 (1 mg/ml)	0.1 ml
$FeSO_4$ (0.4 mg/ml)	0.1 ml
$CaCl_2$ (0.8% w/v)	0.1 ml
$MgCl_2$ (0.1 M)	0.1 ml
Vitamin B12 (0.01 mg/ml)	0.05 ml
L-Cysteine	100 mg
Distilled water	Up to 50 ml total

Solution pH was adjusted to 7.2 and then syringe-filtered through a 0.22 μM membrane filter. It can then be mixed with any 2x carbon source stock (usually 10 mg/ml) in a 1:1 ratio for a 5 mg/ml final carbon source minimal media.
