# Supplementary material for: Identification of d-arabinan-degrading enzymes in mycobacteria
Source: Nat Commun. 2023 Apr 19;14:2233. doi: 10.1038/s41467-023-37839-5 (PMC10115798; doi:10.1038/s41467-023-37839-5)
Supplement: Supplementary file 3 — Description of Additional Supplementary Files [file 41467_2023_37839_MOESM3_ESM.pdf]

## Description of Additional Supplementary Files:

**Supplementary Data 1.** Whole cell proteomics of *D. gadei* grown on D-arabinan, ordered by iBAQ. Proteins from PUL42 are coloured blue. The mass spectrometry data have been deposited to the ProteomeXchange Consortium via the PRIDE partner repository with the dataset identifier PXD039984 (http://www.ebi.ac.uk/pride/archive/projects/PXD039984).
