# Supplementary material for: Identification of d-arabinan-degrading enzymes in mycobacteria
Source: Nat Commun. 2023 Apr 19;14:2233. doi: 10.1038/s41467-023-37839-5 (PMC10115798; doi:10.1038/s41467-023-37839-5)
Supplement: Supplementary file 5 — Reporting Summary [file 41467_2023_37839_MOESM5_ESM.pdf]

nature portfolio

Corresponding author(s): Patrick Moynihan, Elisabeth Lowe

Last updated by author(s): 24/03/2023

Reporting Summary

Nature Portfolio wishes to improve the reproducibility of the work that we publish. This form provides structure for consistency and transparency in reporting. For further information on Nature Portfolio policies, see our Editorial Policies and the Editorial Policy Checklist.

Statistics

For all statistical analyses, confirm that the following items are present in the figure legend, table legend, main text, or Methods section.

n/a	Confirmed	
[ ]	[x]	The exact sample size (*n*) for each experimental group/condition, given as a discrete number and unit of measurement
[ ]	[x]	A statement on whether measurements were taken from distinct samples or whether the same sample was measured repeatedly
[ ]	[x]	The statistical test(s) used AND whether they are one- or two-sided<br>*Only common tests should be described solely by name; describe more complex techniques in the Methods section.*
[ ]	[x]	A description of all covariates tested
[ ]	[x]	A description of any assumptions or corrections, such as tests of normality and adjustment for multiple comparisons
[ ]	[x]	A full description of the statistical parameters including central tendency (e.g. means) or other basic estimates (e.g. regression coefficient) AND variation (e.g. standard deviation) or associated estimates of uncertainty (e.g. confidence intervals)
[ ]	[x]	For null hypothesis testing, the test statistic (e.g. *F*, *t*, *r*) with confidence intervals, effect sizes, degrees of freedom and *P* value noted<br>*Give P values as exact values whenever suitable.*
[x]	[ ]	For Bayesian analysis, information on the choice of priors and Markov chain Monte Carlo settings
[x]	[ ]	For hierarchical and complex designs, identification of the appropriate level for tests and full reporting of outcomes
[x]	[ ]	Estimates of effect sizes (e.g. Cohen's *d*, Pearson's *r*), indicating how they were calculated

Our web collection on statistics for biologists contains articles on many of the points above.

Software and code

Policy information about availability of computer code

Data collection	Detailed methods are in the manuscript, however we used Chromeleon V.6.8 for all HPLC analysis.
Data analysis	Graphpad Prism v8.0.1 or v9.3.1 was used for graphing/plotting/statistical analysis. Proteomics were analysed using MaxQuant version 1.6.12.052. RNASeq was analysed by Rsubread package. IQ-TREE v.1.6.12 was used to generate maximum likelihood phylogenies using automatic model selection and ClustalOmega was used for alignments.

For manuscripts utilizing custom algorithms or software that are central to the research but not yet described in published literature, software must be made available to editors and reviewers. We strongly encourage code deposition in a community repository (e.g. GitHub). See the Nature Portfolio guidelines for submitting code & software for further information.

Data

Policy information about availability of data

All manuscripts must include a data availability statement. This statement should provide the following information, where applicable:

- Accession codes, unique identifiers, or web links for publicly available datasets
- A description of any restrictions on data availability
- For clinical datasets or third party data, please ensure that the statement adheres to our policy

The proteomics dataset was deposited to the ProteomeXchange Consortium via the PRIDE partner repository with the dataset identifier PXD039984 ((http://www.ebi.ac.uk/pride/archive/projects/PXD039984). For RNASeq experiments raw sequencing reads were mapped to Bacteroides cellulosilyticus DSM 14838 (GCA_000158035) downloaded from Ensembl (assembly ID ASM15803v1).

Human research participants

Policy information about studies involving human research participants and Sex and Gender in Research.

Reporting on sex and gender	N/A
Population characteristics	N/A
Recruitment	N/A
Ethics oversight	N/A

Note that full information on the approval of the study protocol must also be provided in the manuscript.

Field-specific reporting

Please select the one below that is the best fit for your research. If you are not sure, read the appropriate sections before making your selection.

- [x] Life sciences
- [ ] Behavioural & social sciences
- [ ] Ecological, evolutionary & environmental sciences

For a reference copy of the document with all sections, see nature.com/documents/nr-reporting-summary-flat.pdf

Life sciences study design

All studies must disclose on these points even when the disclosure is negative.

Sample size	No statistical method was used to pre-determine sample size. For statistical analysis at least three biological replicates were used.
Data exclusions	No data were excluded.
Replication	For all analysis at least 2 technical replicates but typically 3 were used. Replication of the data was successful and no data has been omitted.
Randomization	The experimental design does not require randomization.
Blinding	No blinding was applied due to the nature of these experiments.

Reporting for specific materials, systems and methods

We require information from authors about some types of materials, experimental systems and methods used in many studies. Here, indicate whether each material, system or method listed is relevant to your study. If you are not sure if a list item applies to your research, read the appropriate section before selecting a response.

Materials & experimental systems

n/a	Involved in the study
☒	☐ Antibodies
☒	☐ Eukaryotic cell lines
☒	☐ Palaeontology and archaeology
☒	☐ Animals and other organisms
☒	☐ Clinical data
☒	☐ Dual use research of concern

Methods

n/a	Involved in the study
☒	☐ ChIP-seq
☒	☐ Flow cytometry
☒	☐ MRI-based neuroimaging
